# Circadian Disruption Is Associated with Elevated Whole-Semen mtDNA Copy Number and Implicates *CRY1* as a Candidate Regulator in Humans and Mice

**DOI:** 10.3390/ijms27156569

**Published:** 2026-07-23

**Authors:** Mengchao He, Chuanyu Chen, Jing Gu, Yimeng Wang, Yingzhong Dai, Siwen Luo, Xiaolu Zhao, Baojian Wu, Jia Cao, Qing Chen

**Affiliations:** 1 State Key Laboratory of Trauma and Chemical Poisoning, Key Lab of Medical Protection for Electromagnetic Radiation, Ministry of Education of China, Institute of Toxicology, College of Preventive Medicine, Third Military Medical University (Army Medical University), Chongqing 400038, China; hemengchao@tmmu.edu.cn (M.H.); cyclocytidine@tmmu.edu.cn (C.C.); gj3_26@163.com (J.G.); wym@tmmu.edu.cn (Y.W.); 18571286789@163.com (Y.D.); swluo1999@tmmu.edu.cn (S.L.); zxlulu@tmmu.edu.cn (X.Z.); 2 Institute of Molecular Rhythm and Metabolism, Guangzhou University of Chinese Medicine, Guangzhou 510006, China; bj.wu@hotmail.com

**Keywords:** circadian disruption, social jetlag, semen mitochondrial DNA copy number (mtDNAcn), circadian genes, spermatogenesis

## Abstract

Circadian disruption has been linked to impaired male fecundity, but its association with semen molecular phenotypes and circadian genes remains unclear. We analyzed 441 men from the Male Reproductive Health in Chongqing College Students cohort to assess whether social jetlag, an indicator of circadian disruption, was associated with whole-semen mitochondrial DNA copy number (mtDNAcn), an emerging biomarker of male fecundity. Core circadian genes related to mtDNAcn were screened using genetic polymorphism data. A light-cycle phase-shifting mouse model, *Cry1*-knockout mice, and testicular *Cry1* re-expression models were used for experimental validation, with histology, transcriptomics, single-cell data, and proteomics analyses used to explore mechanisms. Social jetlag was associated with higher mtDNAcn in men (1.29-fold, *p* = 0.026), with a concordant increase in circadian-disrupted mice (1.33-fold, *p* = 0.010). Among core circadian genes, *CRY1* showed the strongest association with mtDNAcn (*p* = 0.048). *Cry1* knockout elevated mtDNAcn (2.18-fold, *p* < 0.001), whereas testicular *Cry1* re-expression reduced it toward wild-type levels. Circadian disruption and *Cry1* deficiency were accompanied by seminiferous epithelial disorganization, spermatogenesis-related transcriptomic changes, and altered mitochondrial pathway signatures. To our knowledge, this study is the first to identify whole-semen mtDNAcn as a circadian-disruption-associated molecular phenotype and supports *CRY1* as a candidate regulator.

## 1. Introduction

Circadian disruption has become a pervasive feature of modern society [1,2,3,4]. The most widespread form of circadian disruption in the population is social jetlag, which refers to the mismatch in sleep timing across different social contexts (for example, workdays versus free days) [5,6]. Epidemiological estimates indicated that average social jetlag in the United States was about 75 min [7] and that roughly 30% of Chinese adults experienced at least 1 h of social jetlag [8]. Accumulating evidence has linked social jetlag to various adverse health outcomes, including impaired semen quality. For example, a study reported that individuals with social jetlag greater than 2 h exhibited a 32.6% reduction in sperm count [9], consistent with findings in zebrafish and mouse models mimicking circadian disruption [10]. However, a previous study has shown that routine semen parameters only contributed about 30% to the male infertility caused by circadian disruption [11], raising the possibility that molecular alterations in semen could provide additional insight.

Semen DNA-related markers have been proposed as sensitive complements to routine semen analysis, particularly given that up to 15% of infertile men present with normal routine parameters [12]. Beyond their correlation with standard sperm metrics, these markers may capture subtle reproductive abnormalities that are missed by conventional semen assessment [12]. Seminal DNA abnormalities include changes involving both nuclear DNA and mitochondrial DNA [13]. The latter is exceptionally vulnerable to environmental insults due to the absence of protective histones and limited DNA repair machinery [14]. During normal spermatogenesis, mature spermatozoa undergo marked mitochondrial remodeling and mtDNA reduction [15], whereas elevated mtDNA content in semen has been linked to abnormal spermatogenesis, impaired mitochondrial quality control, retention of immature germ-cell characteristics, and the release of cell-free mitochondrial material under pathological conditions [16,17,18]. Accordingly, semen-derived mtDNAcn is better interpreted as a composite molecular signal related to disturbed spermatogenesis and altered mitochondrial homeostasis than as a direct readout of mtDNA content in fully mature, healthy spermatozoa; in both clinical and experimental settings, it has therefore been used as a sensitive surrogate biomarker of reproductive impairment [19]. For example, a recent machine learning analysis of 281 men from a general population preconception cohort confirmed that mtDNAcn was the most predictive single molecular measure of couples’ time-to-pregnancy [20]. In animal models, pig sperm with high mtDNAcn showed greater intracellular ROS, reduced motility, and lower in vivo conception rates compared with those with low mtDNAcn, establishing this parameter as a functional fertility marker across species [21]. These findings support the use of whole-semen mtDNAcn as a composite semen-derived molecular indicator that may reflect spermatogenic dysfunction and altered mitochondrial homeostasis, rather than a direct measure of mtDNA content in mature spermatozoa. Biologically, elevated whole-semen mtDNAcn is associated with the cumulative effects of abnormal spermatogenesis [16]. Incomplete germ-cell maturation, impaired cytoplasmic or mitochondrial elimination, oxidative-stress-related mitochondrial injury, compensatory mitochondrial biogenesis, or cell-free mitochondrial material released from damaged cells can all lead to an increase in whole-semen mtDNAcn [16,17,18]. Despite this, whether circadian disruption is associated with altered whole-semen mtDNAcn in humans, and whether such a phenotype can be recapitulated experimentally, remains insufficiently studied.

An additional unresolved question concerns the circadian genes that may contribute to this reproductive phenotype. Core clock genes are known to mediate tissue vulnerability to circadian disruption in several biological systems [22,23], yet the specific circadian components linked to semen molecular abnormalities under circadian disruption exposure remain unclear, especially in human populations.

To bridge these knowledge gaps, the present study integrated evidence from a human cohort and mouse models to examine whether circadian disruption is associated with altered whole-semen DNA-related phenotypes, with a particular focus on mtDNAcn. Subsequently, gene-based SNP analysis, gene-knockout mice, and testicular re-expression mouse models were employed to prioritize circadian gene candidates potentially related to this phenotype. Exploratory omics analyses including single-cell transcriptomics data and proteomic profiling were integrated to explore the underlying mechanisms.

## 2. Results

### 2.1. Circadian Disruption Was Associated with Elevated Whole-Semen mtDNAcn in Men and Mice

**Figure 1:**
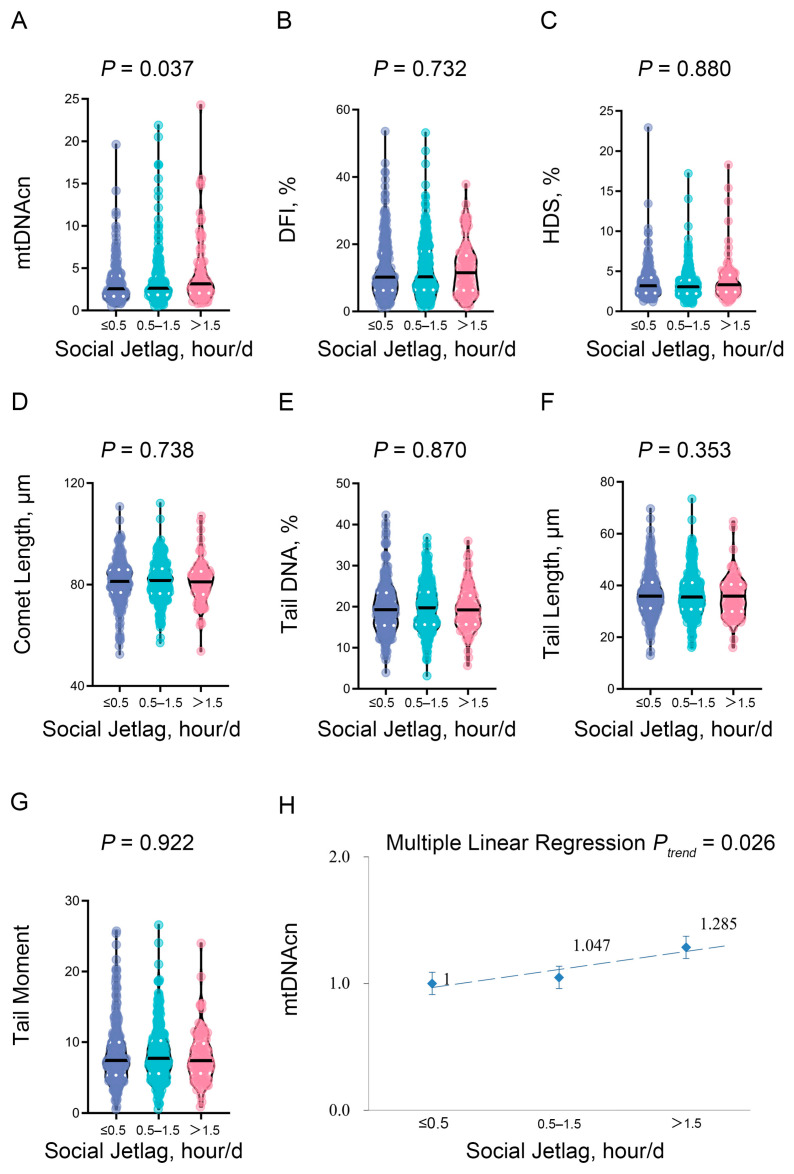
Screening for the sensitive DNA-related marker through population data. (**A**–**G**) Univariate correlation analysis between 7 whole-semen DNA damage parameters and social jetlag. The Jonckheere-Terpstra test was used. (**H**) Multivariate linear regression between mtDNAcn and social jetlag. Adjusted for age, BMI, abstinence days, smoking, alcohol consumption and sleep duration.

Demographic characteristics of the 441 young male participants from the MARHCS cohort are summarized in Table 1. Their mean age was 21 years, and over 15% exhibited social jetlag exceeding 1.5 h. About 45.3% of participants reported sleeping 7–8 h daily. Among the assessed whole-semen DNA damage markers, whole-semen mtDNAcn, but not indicators of whole-semen nuclear DNA, was positively correlated with social jetlag (*p* = 0.037; Figure 1A–G). The significant positive association between social jetlag and mtDNAcn remained robust after adjustment for age, BMI, abstinence period, smoking, alcohol consumption, and sleep duration (*P*_trend_ = 0.026; Figure 1H). Specifically, social jetlag exceeding 1.5 h/day was associated with a 1.29-fold (95% CI: 1.05–1.57) increase in mtDNAcn.

**Figure 2:**
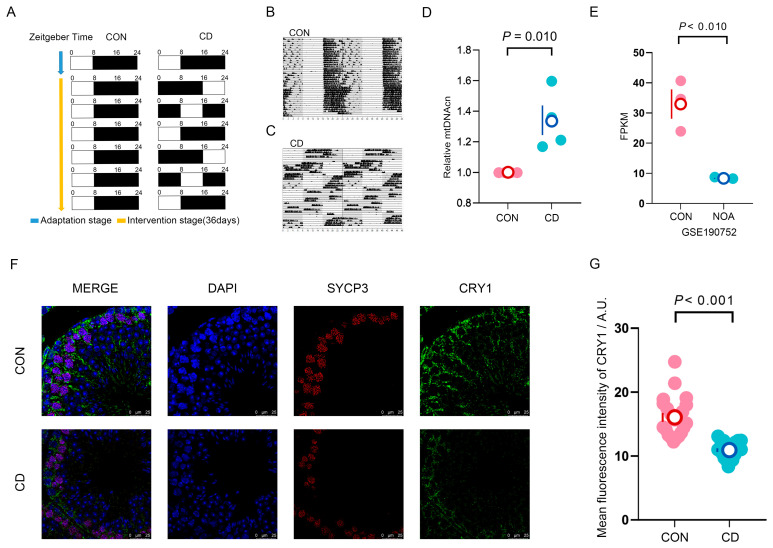
Whole-semen mtDNAcn and CRY1 protein changes in mice exposed to circadian disruption. (**A**) Schematic of intervention in the light-induced CD mouse model. (**B**,**C**) Representative activity plots comparing the control and CD group. (**D**) Relative expression levels of whole-semen mtDNAcn in CD and control mice (*n* = 4 mice). (**E**) *CRY1* expression in human testes from normal controls and NOA patients based on GSE190752 (*n* = 3 patients per group). (**F**) Immunofluorescence staining for CRY1 and SYCP3 in the testis. SYCP3 is a marker for spermatocytes. The scale bar represents 25 μm. (**G**) Quantification of CRY1 fluorescence intensity. Each group measured a total of 21 fields from 3 mice. Abbreviations: mtDNAcn, mitochondrial DNA copy number; CD, circadian disruption; NOA, non-obstructive azoospermia.

To provide experimental support for the epidemiological findings, a mouse model mimicking circadian disruption was established using photoperiod shifting. Following 36 days of shifting light cycles, the experimental group exhibited altered diurnal activity patterns, indicating effective circadian disruption (Figure 2A–C). Mirroring the observation in the MARHCS population, the whole-semen mtDNAcn was significantly elevated by 1.33-fold (*p* = 0.010, Figure 2D) in the circadian disruption group compared to controls.

### 2.2. Exploratory Human Genetic Screening and Mouse Testicular Expression Analysis Prioritized CRY1 for Experimental Evaluation

To explore circadian genes potentially linked to whole-semen mtDNAcn, a gene-based association analysis in the MARHCS cohort was performed as preliminary evidence for prioritization. Within the MARHCS cohort, 13 core circadian genes were identified. Gene-based analysis with MAGMA (Multi-marker Analysis of GenoMic Annotation) revealed that among the preselected core circadian genes, *CRY1* had the lowest nominal *p* value for association with whole-semen mtDNAcn variation (*p* = 0.048, Table 2). To further examine whether *CRY1* was altered in a clinical context of severe spermatogenic failure, we analyzed an independent human testicular transcriptomic dataset from non-obstructive azoospermia (NOA) patients (GEO accession: GSE190752). In this dataset, *CRY1* expression was significantly lower in NOA testes than in normal controls (*p* < 0.010; Figure 2E). Although this external dataset does not establish a causal role for *CRY1*, the concordance between the nominal MAGMA signal and reduced *CRY1* expression in NOA testes supported the testicular relevance of *CRY1* and its prioritization for subsequent experimental modeling.

Given that previous studies suggest that testicular alterations may contribute to semen impairment induced by circadian disruption [9], and that elevated whole-semen mtDNAcn has been proposed to reflect abnormal spermatogenic processes [16], we examined testicular *Cry1* expression in the circadian-disruption mouse model. Immunofluorescence localized *CRY1* predominantly to spermatocytes and spermatogonia within the seminiferous tubules (Figure 2F). Notably, compared to controls, *CRY1* protein levels in the testes of the circadian disruption group were significantly reduced by 32% (*p* < 0.001, Figure 2G).

### 2.3. Cry1 Knockout and Testicular Re-Expression Support a Functional Relationship Between Cry1 and Whole-Semen mtDNAcn

**Figure 3:**
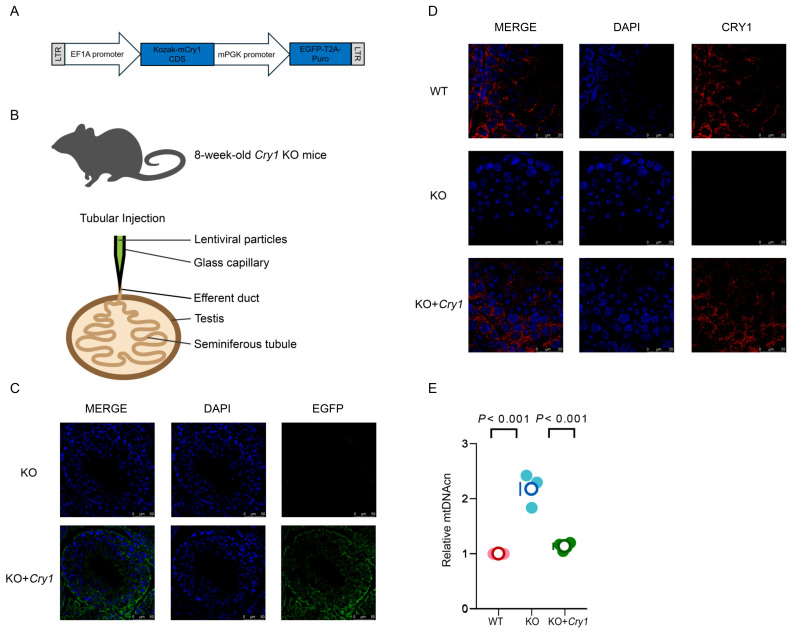
Whole-semen mtDNAcn alteration in *Cry1*-knockout and lentivirus-rescued mice. (**A**) Schematic of the lentivirus vector used in the study. (**B**) Schematic representation of tubular injection of lentivirus. (**C**) Immunofluorescence staining for EGFP in the testis 3 days after injection. The scale bar represents 50 μm. (**D**) Immunofluorescence staining for CRY1 in the testis 3 weeks after injection. The scale bar represents 25 μm. (**E**) Whole-semen mtDNAcn in wild-type, *Cry1*-knockout, and *Cry1*-rescue mice. Data points represent individual animals (*n* = 4 mice per group). Abbreviations: mtDNAcn, mitochondrial DNA copy number; KO, knockout.

To assess whether *Cry1* is functionally relevant to whole-semen mtDNAcn, this study employed global *Cry1*-knockout mice and a testicular lentiviral *Cry1* re-expression strategy (Figure 3A,B). Specifically, *Cry1* lentiviral particles were microinjected into the seminiferous tubules of 8-week-old *Cry1*-knockout mice to restore *Cry1* expression. This approach has been used to restore expression of selected genes in the testis [24]. The EGFP expression detected three days post-injection indicated successful viral delivery (Figure 3C), and immunofluorescence verified CRY1 re-expression in the seminiferous tubules three weeks post-injection (Figure 3D). To encompass a full spermatogenic cycle (~35 days), whole-semen mtDNAcn was assessed six weeks post-injection. Results showed that whole-semen mtDNAcn in *Cry1*-knockout mice was markedly elevated by 2.18-fold compared to age-matched wild-type mice (*p* < 0.001, Figure 3E). Upon testicular *Cry1* re-expression, whole-semen mtDNAcn was significantly reduced compared with age-matched *Cry1*-knockout mice and approached the level observed in wild-type mice (Figure 3E).

### 2.4. Circadian Disruption and Cry1 Deficiency Were Accompanied by Spermatogenesis-Related Histological and Transcriptomic Alterations

**Figure 4:**
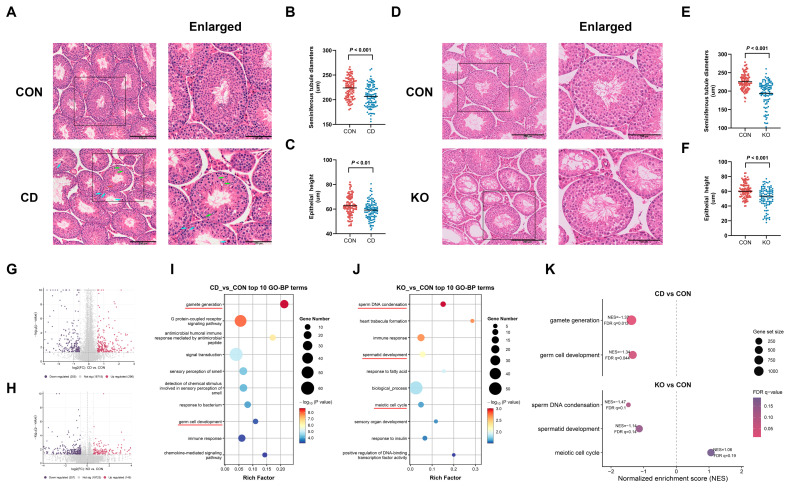
Circadian disruption and *Cry1* deficiency were accompanied by spermatogenesis-related histological and transcriptomic alterations. (**A**) Representative H&E staining in control and CD testes. The green arrows mark vacuolar degeneration changes and nuclear clearing in cells located near the spermatocyte layer, and the blue arrows mark abnormal germ-cell-like elements. (**B**,**C**) Quantification of seminiferous tubule diameter and germinal epithelial height in control and CD testes. Each group measured a total of 100 seminiferous tubules from 3 mice. (**D**) Representative H&E staining in control and *Cry1*-knockout testes. (**E**,**F**) Quantification of seminiferous tubule diameter and germinal epithelial height in control and *Cry1*-knockout testes. Each group measured a total of 100 seminiferous tubules from 3 mice. (**G**,**H**) Volcano plots showing differentially expressed protein-coding genes in CD vs CON and KO vs CON testes. Differentially expressed genes were defined as *p* < 0.05 and |log2FC| ≥ 0.58. (**I**,**J**) GO-BP over-representation analysis of all differentially expressed genes in CD vs CON and KO vs CON testes. The top 10 terms were ranked by *p* value. Spermatogenesis-related pathways are marked with horizontal lines. (**K**) Targeted GSEA of spermatogenesis-related GO-BP terms identified by over-representation analysis. Gene sets with |NES| > 1 and FDR *q*-value < 0.25 were considered supportive. Data are presented as mean ± SEM. Abbreviations: CD, circadian disruption; KO, knockout.

Given that elevated whole-semen mtDNAcn may reflect abnormal spermatogenic progression, we next asked whether CD and *Cry1* deficiency were accompanied by testicular changes related to spermatogenesis. H&E staining showed that testes from CD mice exhibited disturbed seminiferous tubule organization compared with controls, with reductions in seminiferous tubule diameter and seminiferous epithelial height. Morphologically, vacuolar degeneration changes and nuclear clearing were observed in cells located near the spermatocyte layer. The local structure of the spermatogenic cell layer was disordered, and occasional components resembling abnormal germ cells were also visible (Figure 4A–C). In *Cry1*-knockout testes, these alterations were more pronounced, with a subset of seminiferous tubules showing marked epithelial vacuolization and thinning of the seminiferous epithelium (Figure 4D–F). These observations suggest that both CD and *Cry1* loss were associated with altered seminiferous epithelial organization.

We then performed differential expression analysis using protein-coding gene-level transcriptomic data from CD and *Cry1*-knockout testes. In CD testes, 296 genes were upregulated and 255 genes were downregulated compared with controls (Figure 4G). In *Cry1*-knockout testes, 145 genes were upregulated and 257 genes were downregulated compared with controls (Figure 4H). GO-BP over-representation analysis of all differentially expressed genes revealed enrichment of spermatogenesis-related biological processes. In CD testes, the top enriched terms included gamete generation and germ cell development (Figure 4I). In *Cry1*-knockout testes, enriched terms included sperm DNA condensation, spermatid development, and meiotic cell cycle (Figure 4J).

To determine whether these GO-BP terms showed directional enrichment across the full ranked transcriptome, we performed targeted GSEA for the spermatogenesis-related terms identified above. In CD testes, gamete generation showed negative enrichment (NES = −1.37, FDR *q* = 0.012), while germ cell development showed the same direction of enrichment with a weaker FDR signal (NES = −1.34, FDR *q* = 0.044) (Figure 4K). In *Cry1*-knockout testes, sperm DNA condensation (NES = −1.47, FDR q = 0.100) and spermatid development (NES = −1.14, FDR *q* = 0.140) were negatively enriched, whereas meiotic cell cycle showed positive enrichment under the GSEA criterion (|NES| > 1 and FDR *q* < 0.25) (Figure 4K). Together, these histological and transcriptomic findings suggest that CD and *Cry1* deficiency are accompanied by changes consistent with spermatogenesis-related perturbations.

### 2.5. Exploratory Spermatocyte Proteomics Suggested Mitochondrial Pathway Alterations After Cry1 Loss

**Figure 5:**
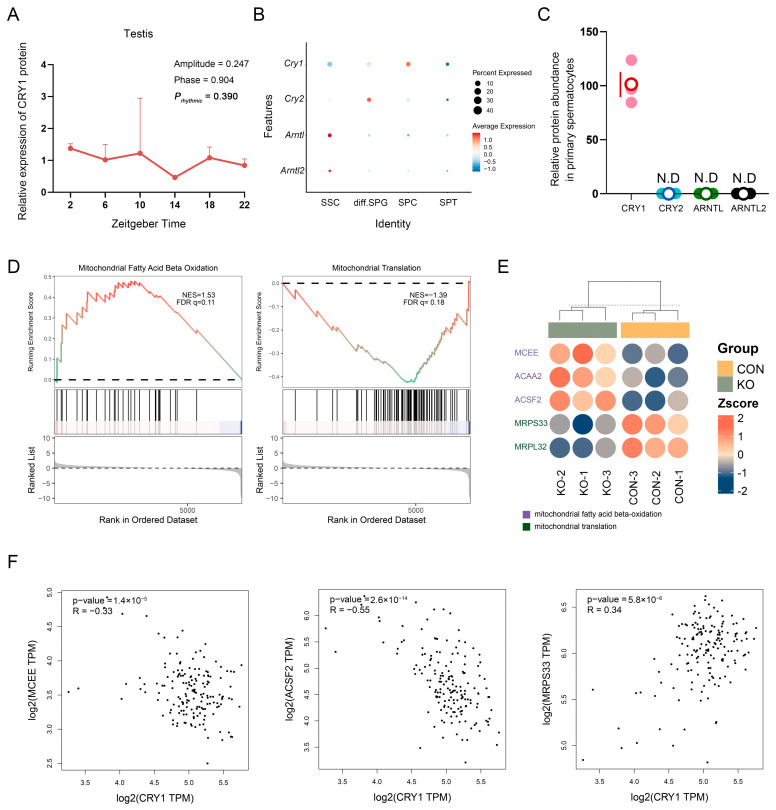
Exploratory proteomic and public-database analyses suggest mitochondrial pathway alterations in *Cry1*-deficient spermatocytes. (**A**) 24-h levels of the CRY1 protein in the testis (*n* = 2 mice per time point). Data from the Mouse Circadian Proteome Atlas. (**B**) Expression patterns of the positive feedback elements within the TTFL, *Arntl* and *Arntl2*, as well as the negative feedback elements, *Cry1* and *Cry2* across cell types during spermatogenesis. Data from the Male Health Atlas. (**C**) Relative protein abundance in the primary spermatocytes (*n* = 3 mice). (**D**) GSEA results from proteomic sequencing of primary spermatocytes in *Cry1* knockout mice and the control group. (**E**) Heatmap of differentially abundant proteins in mitochondrial fatty acid β-oxidation and mitochondrial translation. (**F**) Correlation scatter plots of *CRY1* with *MCEE*, *ACSF2,* and *MRPS33* expression in human testis from the GEPIA database. Abbreviations: SSC, spermatogonial stem cells; diff. SPG, differentiating/differentiated spermatogonia; SPC, spermatocyte; SPT, spermatid; TTFL, transcription-translation feedback loop; N.D, not detected.

Given that the function of circadian clock genes in most tissues is dependent on the transcription-translation feedback loop (TTFL), the spatiotemporal expression pattern of the *Cry1* gene during spermatogenesis was further investigated. Results from multiple time points showed that CRY1 protein expression in the testis did not exhibit rhythmic oscillations (*p* = 0.390, Figure 5A). Subsequently, the cell-type-specific expression of the positive feedback elements within the TTFL, *Arntl* and *Arntl2*, as well as the negative feedback elements, *Cry1* and *Cry2*, during spermatogenesis was further analyzed. The results revealed discordant expression patterns across cell types. Expression of *Arntl* and *Arntl2* was predominantly concentrated in spermatogonial stem cells, whereas *Cry1* and *Cry2* were primarily expressed in spermatocytes and differentiating spermatogonia (Figure 5B). A further stage-resolved analysis using public single-cell RNA-seq data from the Male Health Atlas showed that *Cry1* expression was most evident in pachytene/diplotene spermatocytes and decreased across round spermatid stages, whereas *Arntl* and *Arntl2* were mainly enriched in early spermatogonial populations (Supplementary Figure S2). These results suggested that *Cry1* function in male germ cells may not mirror a classical TTFL configuration. Since both immunofluorescence and single-cell RNA sequencing results indicated that *Cry1* was predominantly expressed in spermatocytes, primary spermatocytes from *Cry1*-knockout and control mice were sorted and subjected to proteomic sequencing. The proteomic sequencing ultimately identified over 80,000 peptides corresponding to more than 7000 proteins. Although CRY1 was detected, its canonical TTFL partners—CRY2, ARNTL, and ARNTL2—were not detected in the proteomic dataset under the present LC-MS/MS sensitivity and sample-processing conditions (Figure 5C). Therefore, together with the publicly available single-cell transcriptomic data, these proteomic results suggested that *CRY1* may act in spermatocytes in a context that does not fully mirror the canonical TTFL configuration observed in many somatic tissues. However, we cannot exclude the possibility that *CRY2*, *ARNTL*, or *ARNTL2* were expressed at low levels but remained below the detection threshold of this exploratory proteomic analysis.

To explore pathways through which *Cry1* may influence mitochondrial biology, the Gene Set Enrichment Analysis (GSEA) was employed. In *Cry1*-knockout spermatocytes, mitochondrial fatty acid beta-oxidation showed positive enrichment (NES = 1.53, FDR *q* = 0.11, Figure 5D), whereas mitochondrial translation showed negative enrichment (NES = −1.39, FDR *q* = 0.18, Figure 5D). Consistent with these findings, levels of MCEE, ACAA2, and ACSF2 showed higher abundance, while MRPS33 and MRPL32 showed lower abundance in *Cry1*-knockout spermatocytes (Figure 5E). To assess whether these signals were directionally compatible with human data, RNA-level correlations of the aforementioned genes in human testicular tissue were analyzed using the GEPIA database. The results showed that *MCEE* (Pearson’s *r* = −0.33, *p* < 0.001, Figure 5F) and *ACSF2* (Pearson’s *r* = −0.55, *p* < 0.001, Figure 5F) were negatively correlated with *CRY1*, while *MRPS33* was positively correlated with *CRY1* (Pearson’s r = 0.34, *p* < 0.001, Figure 5F). These exploratory proteomic and correlation analyses suggest a possible association between *Cry1* loss and altered mitochondrial metabolic pathways in spermatocytes. To further examine whether the mtDNAcn phenotype was more compatible with impaired mitochondrial degradation or increased mtDNA replication, we performed targeted GSEA of mitophagy/mitochondrial-degradation and mtDNA-replication/genome-maintenance pathways using the testicular transcriptomic datasets from *Cry1*-knockout and circadian-disrupted mice. Mitophagy-related pathways showed negative enrichment in both models, whereas mtDNA-replication-related pathways did not show clear enrichment (Supplementary Figure S3). These results suggest that the elevated whole-semen mtDNAcn phenotype is more compatible with altered mitophagy/mitochondrial-degradation signaling than with a clear mtDNA-replication-driven mechanism.

## 3. Discussion

Our integrated analysis of human and mouse experimental data indicated that circadian disruption was associated with elevated whole-semen mtDNAcn in young men and the same directional change was observed in a mouse model of circadian phase shifting. Exploratory human genetic screening, altered testicular *CRY1* expression after circadian disruption, and mouse *Cry1* perturbation experiments together support *CRY1* as a candidate regulator related to this phenotype. Testicular *Cry1* re-expression in *Cry1*-knockout mice reduced the elevated whole-semen mtDNAcn toward wild-type levels, supporting a functional relationship between testicular *Cry1* expression and this semen-derived phenotype. The histological and transcriptomic data showed that circadian disruption and *Cry1* deficiency were accompanied by spermatogenesis-related alterations. Exploratory omics analyses suggested that *Cry1* loss was associated with altered mitochondrial pathway signatures in germ cells, particularly spermatocytes, including pathways related to fatty-acid oxidation and mitochondrial translation.

The present study found that circadian disruption was associated with increased whole-semen mtDNAcn. The whole-semen mtDNAcn has been proposed as a sensitive semen-derived molecular indicator that may precede severe damage to routine semen parameters [19]. Elevated whole-semen mtDNA levels are significantly associated with poorer semen quality [25]. Prior studies have found that circadian disruption exposure leads to reduced sperm count, but whether it affects whole-semen mtDNAcn remains unknown. Our findings extend this literature by suggesting that whole-semen mtDNAcn may capture an additional molecular dimension of circadian-related reproductive vulnerability. Mechanistically, circadian disruption has been reported to perturb cellular redox homeostasis and mitochondrial bioenergetics across tissues, leading to various metabolic diseases [26]. In germ cells and developing spermatogenic lineages, which rely heavily on tightly regulated mitochondrial activity, one possible explanation is that circadian-disruption-related redox imbalance could plausibly affect mitochondrial genome integrity. In the present study, mtDNAcn was quantified from DNA extracted from whole semen rather than density-gradient-purified mature spermatozoa. As noted above, terminally differentiated spermatozoa are known to contain relatively low levels of mtDNA following the spermatogenic mtDNA reduction program [15]. However, it has been proposed in the literature that elevated whole-semen mtDNAcn may reflect the cumulative effects of abnormal spermatogenesis [16]. Several non-mutually exclusive sources may contribute to elevated whole-semen mtDNAcn, including altered spermatogenic progression, retention of immature germ-cell characteristics, abnormal spermatozoa, somatic-cell contribution, or cell-free mitochondrial material [16,27]. At the same time, this phenotype may also reflect a compensatory mitochondrial response to metabolic or oxidative stress, because stress-related mitochondrial injury can trigger mitochondrial biogenesis or altered mtDNA replication through pathways involving TFAM, POLG, and PGC-1α [16,28,29]. These two interpretations are not mutually exclusive: defective maturation or mitochondrial clearance may allow excess or damaged mitochondrial material to persist, while compensatory mitochondrial remodeling may further increase mtDNA-related signals. In the present study, both CD and *Cry1*-deficient testes showed alterations in seminiferous tubule morphology and enrichment of spermatogenesis-related GO-BP terms among differentially expressed genes. Targeted GSEA indicated directional shifts in GO-BP terms identified by over-representation analysis, including gamete generation and germ cell development in CD testes and sperm DNA condensation, spermatid development, and meiotic cell cycle in *Cry1*-knockout testes, which suggested that spermatogenesis-related transcriptional programs are disturbed in both models. The present study also found that *Cry1* loss is accompanied by abnormal mitochondrial signaling in spermatocytes. Therefore, these observations support an association between circadian disruption and elevated whole-semen mtDNAcn, with accompanying testicular changes consistent with spermatogenesis-related perturbation and altered mitochondrial pathway signatures. Beyond characterizing the phenotype, our study implicates *CRY1* as a candidate regulator linked to the whole-semen mtDNAcn phenotype. Previous studies have reported that *Cry1* deficiency provokes testicular dysfunction, with increased germ-cell apoptosis and reduced sperm counts in mice, indicating a direct role for *Cry1* in maintaining testicular homeostasis [30]. In non-spermatogenic cells, NAD^+^ biosynthesis is regulated by *Cry1* through the rhythmic regulation of nicotinamide phosphoribosyltransferase (NAMPT) and related pathways, thereby modulating the activity of mitochondrial sirtuins and protein acetylation. Disruption of *Cry1* function alters NAD^+^ availability and SIRT3-dependent deacetylation of mitochondrial enzymes [31,32]. SIRT3 is a mitochondrial deacetylase that activates key antioxidant and metabolic enzymes by deacetylation, thereby limiting mtROS [33]. When the diurnal oscillation of NAD^+^ and SIRT3-dependent deacetylation programs are altered, the acetylation level of superoxide dismutase 2 increases, thereby reducing mitochondrial antioxidant capacity and elevating mtROS levels [31,34]. However, in germ cells, our results suggested that *Cry1* might regulate mitochondrial function in a non-rhythmic manner independent of the classical TTFL pathway. This observation provides a possible explanation for why *CRY1* showed the strongest nominal signal among the tested core clock genes, although this hypothesis requires validation in larger human cohorts and targeted mechanistic experiments. Additionally, *Cry1* knockout was accompanied by higher signatures of mitochondrial fatty acid β-oxidation and lower signatures of mitochondrial translation in spermatocytes. The upregulation of mitochondrial fatty acid beta-oxidation suggested that increased fatty acid availability was converted to acetyl-CoA, which then entered the tricarboxylic acid (TCA) cycle and oxidative phosphorylation. This might represent a compensatory response by spermatocytes to metabolic stress [35]. Increased mitochondrial fatty acid beta-oxidation could increase electron transport chain flux and thereby potentially promote ROS generation, but direct measurements of mitochondrial respiration and mtROS are needed to test this possibility [36]. This aligns with hepatic evidence that *Cry1* represses lipolysis and beta-oxidation by repressing nuclear receptor pathways such as PPARα [37]. Our finding indicated that this regulation may persist even in spermatocytes, where a canonical TTFL configuration was not evident, highlighting a tissue-specific adaptation of *Cry1* function. Whether similar regulation occurs in spermatocytes requires further confirmation.

Melatonin may represent another biological link between circadian disruption, testicular clock regulation, and mitochondrial oxidative stress. Melatonin secretion is tightly coupled to the light-dark cycle, and inappropriate light exposure at night can suppress or shift nocturnal melatonin secretion [38,39]. Importantly, melatonin is not only an endocrine output of the central circadian system, but also a hormonal signal capable of regulating peripheral clock-gene expression. In the pars tuberalis of rat, melatonin has been shown to induce *Cry1* expression in rats, and melatonin infusion also induces *Cry1* expression in sheep [40,41]. Mechanistically, West et al. reported that melatonin activates *Npas4*, which can transactivate the *Cry1* promoter in the ovine pars tuberalis [42]. Although these studies were not conducted in the testis, they support the broader concept that melatonin can regulate *Cry1*-related clock signaling in peripheral endocrine tissues. More directly relevant to the male reproductive system, melatonin replacement after pinealectomy restored circadian behavior in adult rat Leydig cells and affected the rhythmic expression of clock and steroidogenesis-related genes [43]. A recent study of peripubertal rats also showed that circadian disruption reduced serum melatonin levels and altered the transcriptional pattern and acrophase of core clock genes in Leydig cells, including *Cry1* and *Cry2* [44]. These findings suggest that disrupted melatonin signaling could plausibly contribute to altered testicular clock regulation, including CRY1-related pathways. In the present study, circadian disruption was accompanied by reduced testicular CRY1 expression and elevated whole-semen mtDNAcn, while *Cry1* deficiency produced a similar mtDNAcn phenotype. Therefore, one possible upstream mechanism is that circadian disruption may impair melatonin signaling, disturb testicular clock-gene regulation, and thereby contribute to *CRY1*-related mitochondrial dysregulation. At the same time, this interpretation remains speculative because we did not measure melatonin levels or directly test whether melatonin regulates *Cry1* expression in spermatocytes. Future research can further investigate the role of melatonin.

Despite the concordance between the human and mouse findings, several limitations should be acknowledged. First, the MARHCS cohort had a modest sample size, which limited statistical power, particularly for the MAGMA-based gene-level analysis; therefore, the *CRY1* genetic signal should be interpreted as nominal and hypothesis-generating, which is preliminary evidence for prioritization. Second, the participants were young Chinese men, which may limit generalizability to older men, infertile patients, or populations with different genetic backgrounds. Third, although the observational analyses adjusted for major measured covariates, including sleep duration, residual confounding and exposure misclassification cannot be excluded. For example, social jetlag was assessed using the MCTQ-based questionnaire. Although this index is widely used in population studies, it does not directly capture evening blue-light exposure, dietary timing, meal composition, chrononutrition patterns, or physical activity. These factors may influence the circadian system and mitochondrial metabolism, but they cannot be corrected in this study. In addition, melatonin was not measured in the MARHCS cohort or in the mouse experiments. We therefore cannot determine whether altered melatonin rhythm mediated, modified, or simply accompanied the association between circadian disruption and whole-semen mtDNAcn. Future studies should incorporate dim-light melatonin onset, nocturnal melatonin profiles, or urinary 6-sulfatoxymelatonin together with testicular oxidative-stress and mitochondrial-function measurements. Fourth, whole-semen mtDNAcn was measured from DNA extracted from whole semen rather than purified mature spermatozoa; therefore, this phenotype should be interpreted as a composite semen-derived molecular signal, and contributions from immature germ cells, somatic cells, leukocytes, or cell-free mitochondrial material cannot be formally excluded or quantified. Fifth, although *Cry1* knockout and testicular re-expression support a functional relationship between *Cry1* and whole-semen mtDNAcn, the present study did not directly test whether *Cry1* mediates the effect of circadian disruption on this phenotype. Sixth, the proteomic, transcriptomic, and public-database analyses provide pathway clues but do not define the precise molecular cascade. Although targeted GSEA of testicular transcriptomic data suggested negative enrichment of mitophagy-related pathways rather than clear enrichment of mtDNA-replication-related pathways, the present study did not directly measure mitophagy flux, mitochondrial function, mitochondrial reactive oxygen species (mtROS), mitochondrial DNA damage, or mtDNA replication activity. Meanwhile, in our exploratory study, we cannot rule out the errors caused by the depth of single-cell sequencing and the sensitivity of proteomic sequencing. Targeted validation, such as western blotting or immunofluorescence, will be needed to verify the expression of these proteins in spermatocytes. Seventh, the animal experiments used modest group sizes, especially for several endpoint measurements. Although the direction of the effects was consistent across the circadian-disruption, *Cry1*-knockout, and *Cry1* re-expression models, the magnitude of these effects should be interpreted cautiously until replicated in larger animal cohorts.

In summary, our data show that circadian disruption is associated with elevated whole-semen mtDNAcn, and that exploratory human genetic screening together with mouse *Cry1* perturbation experiments support *CRY1* as a candidate regulator related to this phenotype. Rather than defining a direct increase in mtDNA content of healthy mature sperm, the present study identifies a reproducible semen-derived molecular signal associated with circadian disruption and consistent with disturbed spermatogenesis and altered mitochondrial homeostasis. These findings provide a focused, hypothesis-generating framework for future studies on *CRY1*, semen-derived mitochondrial signals, and circadian-disruption-related male reproductive dysfunction.

## 4. Materials and Methods

### 4.1. Ethics Approval

The protocol for the human study was approved by the Ethics Committee of the Third Military Medical University (approval no. 1.0/2013.4.12). All participants enrolled voluntarily and signed written informed consent before any study procedures. Animal work was approved by the Institutional Animal Care and Use Committee of Army Medical University (approval no. AMUWEC20226243) and was carried out under the approved protocol. Animal numbers and procedures were planned to limit avoidable pain, distress, and use of animals.

### 4.2. Study Population

This work was based on a longitudinal cohort of male university students, the Male Reproductive Health in Chongqing College Students (MARHCS) study, conducted between June 2013 and June 2015. The overall design and baseline characteristics of MARHCS have been described in detail elsewhere [45]. For the present analysis, data from the 2014 follow-up visit were used, including 441 eligible participants. Each man completed a standardized questionnaire collecting information on demographic and lifestyle factors (including social jetlag), underwent a physical examination, and provided semen for sperm analysis and peripheral blood specimens for whole-genome genotyping.

### 4.3. Assessment of Social Jetlag

Social jetlag, an indicator of environmental circadian disruption, was evaluated at each survey using the validated Chinese version of the Munich Chronotype Questionnaire (MCTQ) [9]. For each participant, midsleep was calculated as the midpoint between self-reported sleep onset and wake-up time, separately for study (school) days and free (non-school) days. Social jetlag was calculated as the absolute difference between the midsleep time of free days and that of study days.

### 4.4. Assessment of Whole-Semen mtDNAcn in Humans and Mice

Whole-semen mitochondrial DNA copy number (mtDNAcn) was quantified by real-time quantitative PCR using DNA extracted from whole-semen samples, following published methods with minor adaptations [46,47]. Briefly, DNA was isolated from human whole-semen samples and mouse semen-derived samples using the E.Z.N.A.^®^ DNA/RNA Kit (R6731-01, Omega Bio-tek, Norcross, GA, USA) according to the manufacturer’s protocol. Samples were lysed in GTC Lysis Buffer containing β-mercaptoethanol and then transferred to a HiBind^®^ DNA Mini Column. After DNA binding, column washing with HBC Buffer and DNA Wash Buffer, and column drying, DNA was eluted with Elution Buffer and stored at −20 °C until analysis. DNA concentration and purity were assessed before qPCR. Quantitative PCR was performed on a QuantStudio 3 Real-Time PCR System (Applied Biosystems, Thermo Fisher Scientific, Waltham, MA, USA) using 2X Universal SYBR Green Fast qPCR Mix (RK21203, ABclonal, Wuhan, China). The assay estimated relative mtDNAcn by comparing amplification of a mitochondrial target gene with that of a nuclear reference gene. For human samples, the mitochondrial target was 16S rRNA and the nuclear reference gene was GAPDH. For mouse samples, the mitochondrial target was mt-Nd1 and the nuclear reference gene was Rn18s. Primer sequences were as follows. Human 16S rRNA: forward 5′-ACTTTGCAAGGAGAGCCAAA-3′, reverse 5′-TGGACAACCAGCTATCACCA-3′. Human GAPDH: forward 5′-GGATGATGTTCTGGAAGAGCC-3′, reverse 5′-AACAGCCTCAAGATCATCAGC-3′. Mouse mt-Nd1: forward 5′-TCCGAGCATCTTATCCACGC-3′, reverse 5′-GTATGGTGGTACTCCCGCTG-3′. Mouse Rn18s: forward 5′-GCGTTATTCCCATGACCCG-3′, reverse 5′-CTGTCAATCCTGTCCGTGTCC-3′. Each 20 μL PCR reaction contained 10 ng genomic DNA and was run in triplicate. Cycling conditions for both genes were 50 cycles of 95 °C for 15 s and 60 °C for 15 s, followed by a melting-curve analysis to verify product specificity. A pooled DNA sample prepared from 20 randomly selected participants was included on every run as an internal standard to correct for inter-assay variation. Relative mtDNAcn was calculated using the 2^−ΔΔCt^ method as the ratio of the mitochondrial target gene to the nuclear reference gene and normalized to the internal standard where applicable. Because DNA was extracted from whole semen rather than purified mature spermatozoa, the measured mtDNAcn represents a whole-semen composite signal and should not be interpreted as mature sperm-specific mtDNA content.

### 4.5. Assessment of Whole-Semen Nuclear DNA Damage Markers

Whole-semen nuclear DNA fragmentation was assessed using the sperm chromatin structure assay (SCSA) and the comet assay, following our previously described protocol [46,48]. For SCSA, semen aliquots were thawed, diluted in TNE buffer to 2 × 10^6^ cells/mL, and exposed to an acid-detergent solution to denature DNA at sites of strand breakage. Cells were then stained with acridine orange (AO), which fluoresces green when bound to native double-stranded DNA and red when bound to denatured single-stranded DNA. Within 5 min after AO staining, at least 10,000 sperm per sample were analyzed on an FC500 flow cytometer (Beckman Coulter, Brea, CA, USA). Green (530 ± 30 nm) and red (>630 nm) fluorescence were recorded. The DNA fragmentation index (DFI) was calculated as red fluorescence divided by total fluorescence (high red/low green) and served as an indirect indicator of DNA strand breaks. High DNA stainability (HDS) sperm (high green/low red) is interpreted as a marker of incomplete chromatin condensation or nuclear immaturity.

Comet assay was further performed by the following protocol, as previously reported [46]. Sperm suspensions (4 × 10^6^ cells/mL) were mixed with low-melting-point agarose and layered onto slides pre-coated with normal-melting agarose. After solidification, the slides were immersed in lysis buffer and incubated with DNase-free proteinase K at 37 °C for 16 h to remove membranes and most proteins. Slides were then equilibrated in alkaline electrophoresis buffer to allow DNA unwinding and subjected to electrophoresis at 4 °C. After neutralization and fixation, DNA was stained with ethidium bromide (20 μg/mL). Comet images were captured under a fluorescence microscope (ECLIPSE TE2000-S, Nikon, Tokyo, Japan), and quantitative parameters were obtained using CASP software (version 1.2.2). Comet length, tail DNA, tail length, and tail moment were selected to reflect the levels of DNA damage in single sperm, as supplements to the SCSA results.

### 4.6. Assessment of Potential Confounders

Potential confounders were selected based on the previous literature [9,49]. These confounders included age (continuous variable), body mass index (BMI) (continuous variable), smoking (non-smoker, former smoker, current smoker), alcohol consumption (non-drinkers, former drinkers, current drinkers), and abstinence duration (continuous variable). These potential confounders were measured using a standard questionnaire as the baseline. Additionally, sleep duration was assessed using the Munich Chronotype Questionnaire (MCTQ) [50,51]. In the present study, sleep duration was set as a nominal variable (≤6 h, 6–7 h, 7–8 h, 8–9 h, >9 h) during multivariate analyses.

### 4.7. Genome-Wide Genotyping and Quality Control

Peripheral blood was collected, and genomic DNA was extracted from nucleated cells. Genome-wide SNP genotyping was performed using the Infinium Global Screening Array-24 v1.0 BeadChip (Illumina, San Diego, CA, USA). Quality control of raw genotype data was conducted in PLINK v1.9. Variants with a call rate < 80% were excluded, as were individuals with >2% missing genotypes or discordant genetic and recorded sex. SNPs with minor allele frequency (MAF) < 0.05, deviation from Hardy–Weinberg equilibrium (*p* < 1 × 10^−4^), or low call rate were removed. Individuals whose autosomal heterozygosity deviated by more than ±3 standard deviations from the mean were excluded as potential outliers. Imputation was performed using the phase I whole-genome sequencing reference panel from the China Metabolic Analytics Project (ChinaMAP). Genotypes were aligned to GRCh38/hg38, sorted, and split by chromosome. Haplotype phasing was conducted with Eagle2, followed by SNP imputation with Minimac4 using default parameters. After imputation, chromosomal datasets were merged. Variants were retained if they had an imputation INFO score > 0.7, MAF ≥ 0.001, and call rate > 99%, yielding 8,519,784 high-quality autosomal SNPs for downstream analyses.

### 4.8. Screen for Circadian Genes with Multi-Marker Analysis of GenoMic Annotation (MAGMA)

We prespecified 13 core circadian genes. SNPs located within each gene and 2 kb upstream or downstream were extracted from the imputed dataset. Of 3340 candidate variants, 2595 remained after application of the genome-wide quality-control criteria. Gene-level associations with whole-semen mtDNAcn were then tested in MAGMA [52].

### 4.9. Mouse Model of Circadian Disruption Exposure

Male SPF C57BL/6J mice (8–10 weeks of age) were used because the study outcome concerned male reproductive and semen-derived molecular phenotypes; therefore, sex was not analyzed as an independent biological variable. Mice were housed in cages equipped with running wheels (Probcare, Wuhan, China) placed inside light-tight cabinets (20–22 °C, 50–70% humidity) under an 8L:16D schedule unless otherwise indicated, with approximately 200 lux during the light phase and ad libitum access to food and water. Twelve mice were entrained for two weeks and were then randomly allocated in equal numbers to control and circadian-disruption groups. Computer-generated random numbers were used to generate the randomized sequence. Sample sizes were determined based on prior studies using similar animal models, experimental feasibility, and the 3Rs principle to reduce animal use [9]. The control group maintained the 8L:16D schedule, whereas the experimental group underwent an 8-h light-dark-cycle advance every 6 days for 36 days. Wheel-running activity was recorded in 1-min bins, and actograms and rest-activity rhythms were analyzed using ClockLab (Actimetrics, Wilmette, IL, USA).

### 4.10. Source of Cry1 Knockout Mice

Male SPF *Cry1*-knockout mice on a C57BL/6 background were provided by co-author Baojian Wu. This mouse line was originally obtained from Cyagen Biosciences (Guangzhou, China) and had been validated in a previous peer-reviewed study by Lin et al. [53]. The *Cry1*-knockout line carries a deletion of exon 2 of *Cry1*. In the published validation, PCR-based genotyping was performed using mouse tail DNA and primers targeting the wild-type and deleted *Cry1* alleles. *Cry1* knockout mice showed a 769-bp PCR fragment, whereas wild-type mice showed a 761-bp fragment. The qPCR analysis further confirmed the absence of wild-type *Cry1* transcript in *Cry1* knockout mice. In addition, Dbp and Rev-erbα, two BMAL1/CLOCK target genes normally repressed by *CRY* proteins, were significantly upregulated in *Cry1* knockout mice, supporting functional disruption of *CRY*-mediated transcriptional repression. In the present study, age-matched SPF C57BL/6J male mice supplied by the Experimental Animal Center of Army Medical University served as wild-type controls.

### 4.11. Lentiviral Packaging and Transfection

Recombinant lentiviral vectors for *Cry1* overexpression were designed and synthesized by Cyagen Biosciences (Guangzhou, China). Briefly, the complete coding sequence (CDS) of the mouse *Cry1* gene (NM_007771.4, 1830 bp), flanked by a Kozak consensus sequence, was cloned into a lentiviral transfer expression vector. The resulting plasmid, LV-EF1A > Kozak-Mouse *Cry1* CDS-mPGK > EGFP, contained an EF1A promoter driving the *Cry1* CDS, alongside a murine phosphoglycerate kinase (mPGK) promoter driving an enhanced green fluorescent protein (EGFP). An empty vector without the *Cry1* CDS (LV-mPGK > EGFP) was used as a negative control. Lentiviral particles were packaged by co-transfecting HEK-293T cells with the specific transfer plasmid, the psPAX2 packaging plasmid, and the pMD2.G envelope plasmid using Lipofectamine 3000 (Invitrogen, USA). Viral supernatants were harvested at 48 h and 72 h post-transfection, filtered through a 0.45-μm PVDF membrane, concentrated via ultracentrifugation, and resuspended in sterile phosphate-buffered saline (PBS) for downstream applications.

### 4.12. Injection of Lentiviral Particles

To restore *Cry1* expression in vivo, direct microinjection of lentiviral particles into the seminiferous tubules of *Cry1*-knockout mice was performed as previously described [54]. Eight-week-old male SPF *Cry1*-knockout mice were anesthetized with Avertin. Before use, Avertin was diluted with sterile normal saline to prepare a 2.5% working solution and administered at 300 μL per mouse. Adequate anesthesia was confirmed before surgery by the loss of pedal withdrawal reflex. The testes were gently exposed from the abdominal cavity under aseptic conditions. Approximately 7 μL of concentrated lentiviral suspension, mixed with 0.3% trypan blue, was carefully injected into the efferent duct using a glass capillary needle under a stereomicroscope. An equal volume of control lentivirus was injected into the contralateral testis. After blue dye was observed to fill the seminiferous tubules, the testes were returned to the scrotal sac and the incisions were sutured. After surgery, mice were placed in a 37 °C recovery environment until they regained consciousness and were monitored for postoperative recovery according to the approved animal protocol. EGFP and CRY1 detection in the testes was performed 3 and 21 days after injection, respectively, while whole-semen mtDNAcn analysis was assessed 6 weeks after injection.

### 4.13. Mouse Sample Collection and Euthanasia

At each experimental endpoint, trained staff euthanized the mice by cervical dislocation under the approved animal protocol. Death was confirmed before tissues were collected. Cauda epididymides were obtained for semen-derived mtDNAcn measurement, and testes were processed for histology, immunofluorescence, transcriptomics, or proteomics according to the relevant experiment.

### 4.14. Immunofluorescence Staining

Testicular tissue was fixed, embedded in paraffin, and sectioned into 5 μm sections. The paraffin sections were deparaffinized in xylene and then rehydrated using a series of ethanol gradients. The sections were then fully immersed in EDTA buffer (pH 9.0) and heated in a water bath for 16 min. After cooling to room temperature and washing with phosphate-buffered saline (PBS), the sections were blocked with goat serum at room temperature for 30 min. Subsequently, the sections were incubated overnight at 4 °C with the following primary antibodies: anti-CRY1 (1:200; Proteintech, 13474-1-AP), anti-DDX4 (1:400; Proteintech, 67147-2-Ig), and anti-SYCP3 (1:200; Abcam, ab97672). After washing three times with PBS, the sections were incubated with secondary antibodies, including Alexa Fluor 488-labeled goat anti-rabbit IgG (1:500; Invitrogen, A11008), Alexa Fluor 568-labeled goat anti-rabbit IgG (1:500; Invitrogen, A11011), and Alexa Fluor 568-labeled goat anti-mouse IgG (1:500; Invitrogen, A11004), for 1 h at 37 °C in the dark. Finally, after repeated washing, the sections were mounted using ProLong™ Gold anti-fade mountant containing DAPI (Invitrogen, P36931). Immunofluorescence images were acquired using a laser confocal microscope (Leica, SP8).

### 4.15. Testicular Histology and Morphometric Analysis

Testes from control, CD, and *Cry1*-knockout mice were fixed in 4% paraformaldehyde for 24 h, paraffin embedded, cut at 5 μm, and stained with H&E. Images were acquired with the same microscope settings for all groups. Morphometry was restricted to round or near-round tubule profiles to reduce bias from oblique sectioning. Seminiferous tubule diameter and germinal epithelial height were measured in ImageJ V1.8.0.

### 4.16. Isolation of Mouse Primary Spermatocytes Using Fluorescence-Activated Cell Sorting

Testicular cell suspensions were prepared from fresh adult mouse testes as previously reported [55,56,57]. Briefly, the tunica albuginea was removed, and seminiferous tubules were mechanically dissociated into a single-cell suspension using 25 mg/mL collagenase type IV (Sigma, St. Louis, MO, USA) and 400 U/mL DNase I (Sigma) for 5 min at 35 °C in DMEM: F12. The resulting seminiferous tubules were enzymatically digested in DMEM/F12 medium supplemented with 200 µg/mL trypsin and 400 U/mL DNase I. The digestion was performed at 35 °C with constant agitation (215 rpm) for 4 min and terminated by the addition of fetal bovine serum (FBS). The cell suspension was sequentially filtered through 40-μm strainers. The cells were stained with Hoechst 33,342 (Invitrogen, Carlsbad, CA, USA) and Dye 780 (62910-00, Invitrogen, CA, USA) for 30 min at room temperature in the dark. The work solution was prepared according to the manufacturer’s instructions. After staining, the suspension was filtered again through a 40-μm strainer. The gating method for flow cytometry is shown in Supplementary Figure S1 and is described in the previous literature [55]. The primary spermatocyte populations were identified and sorted based on 4C DNA content using the Sony SH800Z cell sorter. Sorted spermatocytes were collected into tubes containing DMEM supplemented with 10% fetal bovine serum for downstream applications.

### 4.17. Proteomic Analysis

Majorbio Bio-Pharm Technology Co., Ltd. (Shanghai, China) performed DIA proteomic analysis on the sorted primary spermatocytes. Cells were lysed in buffer containing 8 M urea, 1% SDS, and protease inhibitors. Lysates were sonicated on ice and centrifuged at 12,000× *g* for 20 min at 4 °C. The soluble fraction was retained, and its protein concentration was measured before digestion. For tryptic digestion, proteins were reduced with tris (2-carboxyethyl) phosphine (TCEP), alkylated with iodoacetamide (IAA) in the dark, and digested overnight at 37 °C with trypsin. The resulting peptides were desalted, vacuum-dried, and reconstituted for LC-MS/MS analysis.

Peptides were analyzed using a VanquishNeo UHPLC system coupled to an Orbitrap Astral mass spectrometer (Thermo Fisher Scientific, USA). Chromatographic separation was performed on a uPAC High Throughput column (75 μm × 5.5 cm; Thermo Fisher Scientific, USA) with solvent A consisting of water containing 2% acetonitrile and 0.1% formic acid and solvent B consisting of water containing 80% acetonitrile and 0.1% formic acid. The chromatography run time was 8 min. Data were acquired in data-independent acquisition (DIA) mode using Thermo Xcalibur 4.7 software, with the Orbitrap Astral mass spectrometer operated in positive-ion mode, an ion-source voltage of 1.5 kV, and a mass scan range of 100–1700 m/z. Protein identification and quantification were performed using Spectronaut™ 19 software. The main search parameters included trypsin/P digestion, up to two missed cleavages, carbamidomethylation of cysteine as a fixed modification, oxidation of methionine and protein N-terminal acetylation as variable modifications, and protein and peptide false discovery rates controlled at <1%. Protein quantification was performed using MaxLFQ.

### 4.18. Testicular Transcriptomic Analysis and GO Enrichment

Testicular transcriptomic data from CD and control mice, as well as from KO and control mice were reanalyzed using the gene-level expression matrix generated from the sequencing project. Each group contained 3 mouse testicular samples. After filtering non-protein-coding entries, protein-coding genes were retained for downstream analysis.

Read-count matrices were analyzed with DEGseq and DESeq2. Differential expression was defined as *p* < 0.05 with |log2 fold change| ≥ 0.58, and the results were displayed in volcano plots. Differentially expressed genes from each comparison were then entered into GO biological-process over-representation analysis using the modified Fisher exact test. Terms were ordered by *p* value, and the ten highest-ranked terms were plotted as −log10(*p*).

### 4.19. Gene Set Enrichment Analysis (GSEA) and Differential Protein Analysis

Gene Set Enrichment Analysis (GSEA) was performed using the aforementioned testicular transcriptomic datasets and primary-spermatocyte proteomic dataset. Genes or proteins were ranked by the direction and strength of group differences using sign(log2FC) × −log10(*p* value). Gene sets were obtained from the Gene Ontology (GO), Reactome, and KEGG databases. For the targeted analysis related to mitochondrial degradation and mtDNA replication, gene sets associated with mitophagy, regulation of mitophagy, PINK1/PRKN-mediated mitophagy, mitochondrial DNA replication, mitochondrial genome maintenance, and mitochondrial biogenesis were examined. Pathways with an absolute normalized enrichment score (|NES|) > 1 and a false discovery rate (FDR) *q* value < 0.25 were considered enriched.

### 4.20. Analysis of Data from Public Databases

Data on the diurnal rhythm of the CRY1 protein in the testis were obtained from the Mouse Circadian Proteome Atlas (https://chronoproteinology.org/circadian_atlas, 1 April 2026). Single-cell transcriptomic sequencing data from germ cells were obtained from the Male Health Atlas (http://malehealthatlas.cn/, 1 April 2026) in order to identify the cell types where the different circadian genes showed their peak of expression. Gene correlation data for human testicular tissue were obtained from the GEPIA database (http://gepia.cancer-pku.cn/, 1 April 2026), where Pearson correlation was used for correlation analysis. The expression of *CRY1* in human testes from normal controls and non-obstructive azoospermia (NOA) patients was examined using the public GEO dataset GSE190752 [58]. *CRY1* expression values were extracted as FPKM and compared between groups using an unpaired two-sided Student’s *t*-test.

### 4.21. Statistical Analysis

Continuous variables for human participants are summarized as mean ± standard deviation; categorical variables as number (percentage). Associations of social jetlag with whole-semen nuclear and mitochondrial DNA indices were first explored using univariate linear regression and the Jonckheere–Terpstra trend test, then modeled with multivariable linear regression adjusted for age, BMI, abstinence period, smoking status, alcohol consumption, and sleep duration. The mtDNAcn was log10-transformed; regression estimates were back-transformed for interpretation. These statistical analyses were performed using IBM SPSS Statistics, version 26.0. Two-sided *p* values < 0.05 were considered statistically significant. For the animal experiments, formal normality testing was not performed because of the small group sizes. Data distributions were inspected graphically where applicable. The comparisons between control and exposed groups were conducted using Student’s *t*-test in GraphPad Prism, version 10.0. The R package “circacompare” was used to analyze diurnal rhythms using R version 4.5.

## Figures and Tables

**Table 1:** Characteristics of the study population.

Variables	Values
Demographics	
Age, years (*n* = 441)	21.41 ± 1.14
BMI, kg/m^2^ (*n* = 438)	21.56 ± 2.60
Abstinence period, days (*n* = 441)	4.28 ± 1.40
Tobacco smoking (*n* = 440)	
Never	315 (71.6%)
Current	103 (23.4%)
Quit	22 (5.0%)
Alcohol drinking (*n* = 441)	
Never	85 (19.3%)
Current	334 (75.7%)
Quit	22 (5.0%)
Sleep duration, hours/day (*n* = 422)	
≤6 h	8 (1.9%)
6–7 h	70 (16.6%)
7–8 h	191 (45.3%)
8–9 h	119 (28.2%)
>9 h	34 (8.0%)
Social jetlag, hours/day (*n* = 441)	
Median (IQR)	0.75 (0.25–1.25)
≤0.5	170 (38.6%)
0.5–1.5	201 (45.6%)
>1.5	70 (15.8%)
Sperm nuclear DNA parameters	
DFI, % (*n* = 436)	12.93 ± 8.42
HDS, % (*n* = 436)	3.59 ± 2.12
Tail DNA, % (*n* = 438)	19.77 ± 5.94
Tail length, μm (*n* = 438)	36.64 ± 8.31
Comet length, μm (*n* = 438)	81.34 ± 8.13
Tail moment (*n* = 438)	8.28 ± 4.02
Sperm mitochondrial DNA parameters	
mtDNAcn (*n* = 441)	3.68 ± 3.24

Data were presented as mean ± standard deviation (SD), median (interquartile range, IQR), or no. (%), as appropriate.

**Table 2:** Association of the core circadian genes with whole-semen mtDNAcn: MAGMA analysis.

Gene	Chr	Position (GRCh38)	*p* Value
*CRY1*	12	106,991,364–107,093,857	0.048
*CRY2*	11	45,847,118–45,883,248	0.405
*ARNTL*	11	13,277,727–13,387,811	0.057
*CLOCK*	4	55,427,901–55,546,909	0.435
*PER1*	17	8,140,470–8,156,405	0.274
*PER2*	2	238,244,038–238,290,102	0.451
*PER3*	1	7,784,380–7,845,181	0.140
*NR1D1*	17	40,092,784–40,100,725	0.682
*NR1D2*	3	23,945,260–23,980,618	0.577
*NPAS2*	2	100,820,151–100,996,827	0.057
*RORA*	15	60,488,284–61,229,303	0.063
*RORB*	9	74,497,336–74,687,201	0.902
*RORC*	1	151,806,071–151,832,238	0.213

Abbreviations: mtDNAcn, mitochondrial DNA copy number; MAGMA, Multi-marker Analysis of GenoMic Annotation; Chr, chromosome.

## Data Availability

The original contributions presented in this study are included in the article/supplementary material. Further inquiries can be directed to the corresponding authors.

## Supplementary Materials

The following supporting information can be downloaded at: https://www.mdpi.com/article/10.3390/ijms27156569/s1.

## Abbreviations

The following abbreviations are used in this manuscript:

mtDNAcn	mitochondrial DNA copy number
MARHCS	the Male Reproductive Health in Chongqing College Students
MCTQ	the Munich Chronotype Questionnaire
DFI	the DNA Fragmentation Index
HDS	high DNA stainability
MAGMA	Multi-marker Analysis of GenoMic Annotation
