# Supplementary material for: Circadian Disruption Is Associated with Elevated Whole-Semen mtDNA Copy Number and Implicates CRY1 as a Candidate Regulator in Humans and Mice
Source: Int J Mol Sci. 2026 Jul 23;27(15):6569. doi: 10.3390/ijms27156569 (PMC13465147; doi:10.3390/ijms27156569)
Supplement: Supplementary file 1 [file ijms-27-06569-s001.zip › ijms-4355555-supplementary.pdf]

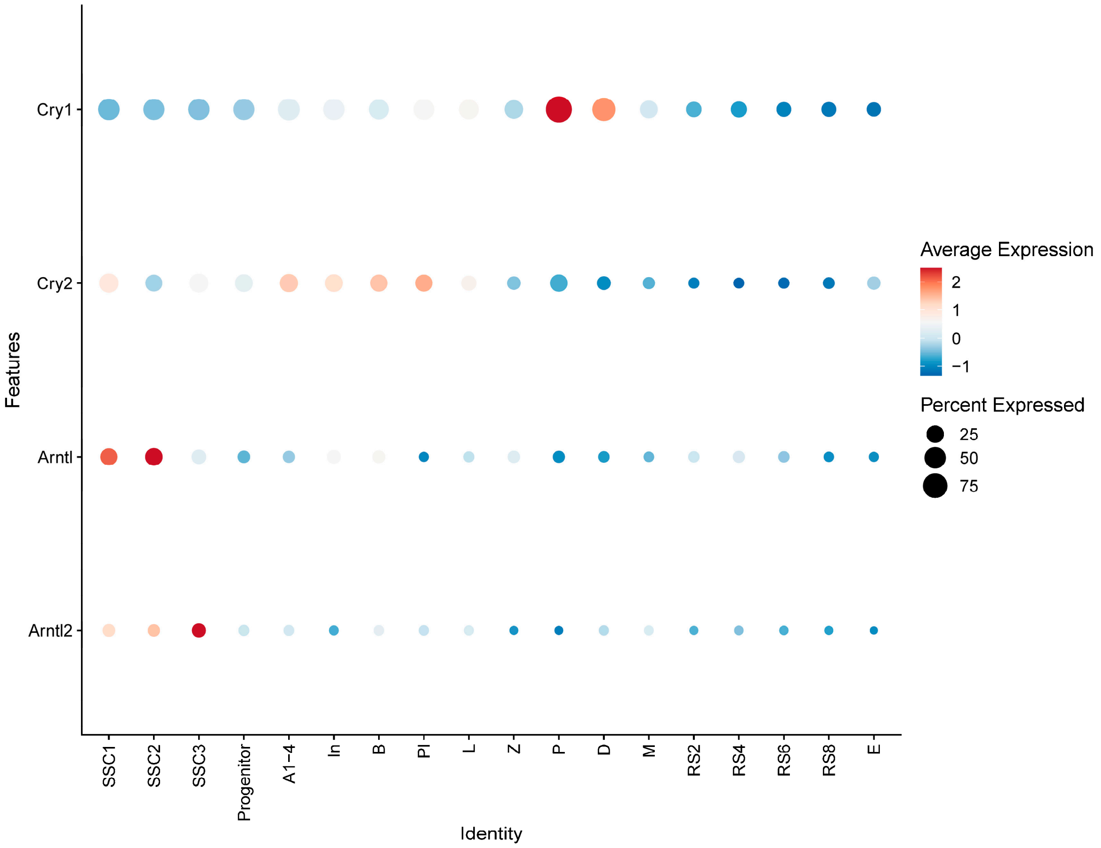

Supplementary Figure S1. Stage-resolved expression of *Cry1*, *Cry2*, *Arntl*, and *Arntl2* during spermatogenesis based on public single-cell RNA-seq data from the Male Health Atlas. Dot color represents scaled average expression, and dot size represents the percentage of cells expressing each gene. Abbreviations: SSC1, spermatogonial stem cell I; SSC2, spermatogonial stem cell II; SSC3, A-aligned spermatogonia III; Progenitor, spermatogonial progenitor cell; A1–4, A1–A4 spermatogonia; In, intermediate spermatogonia; B, type B spermatogonia; Pl, preleptotene spermatocyte; L, leptotene spermatocyte; Z, zygotene spermatocyte; P, pachytene spermatocyte; D, diplotene spermatocyte; M, metaphase spermatocyte; RS, round spermatid; E, elongating spermatid.

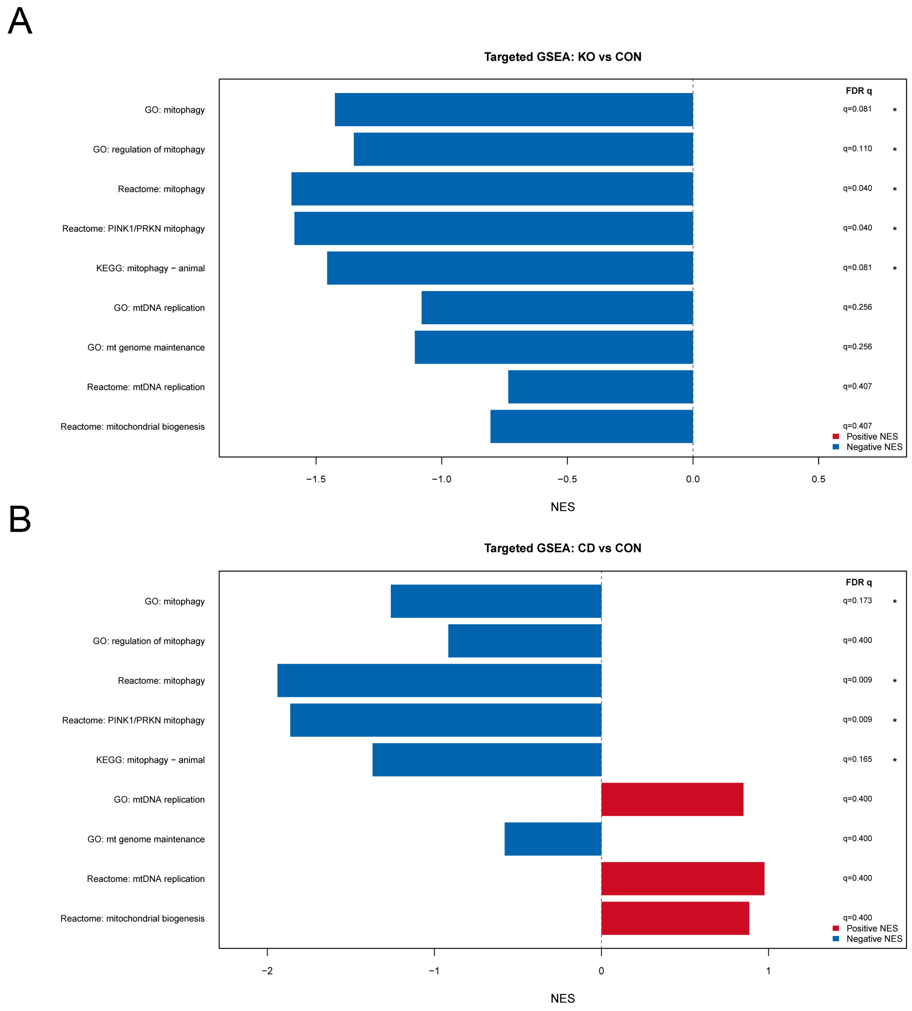

Supplementary Figure S2. Targeted GSEA of mitophagy/mitochondrial-degradation and mtDNA-replication/genome-maintenance pathways in testicular transcriptomic datasets from *Cry1*-knockout and circadian-disrupted mice. Bars indicate the normalized enrichment score (NES). Blue and red bars indicate negative and positive enrichment, respectively. FDR *q* values are shown on the right. Asterisks indicate pathways meeting the predefined enrichment criterion (|NES| > 1 and FDR *q* < 0.25). (A) Targeted GSEA of KO and CON group. (B) Targeted GSEA of CD and CON group. Abbreviations: CD, circadian disruption; KO, *Cry1* knockout; CON, control; NES, normalized enrichment score; FDR, false discovery rate.

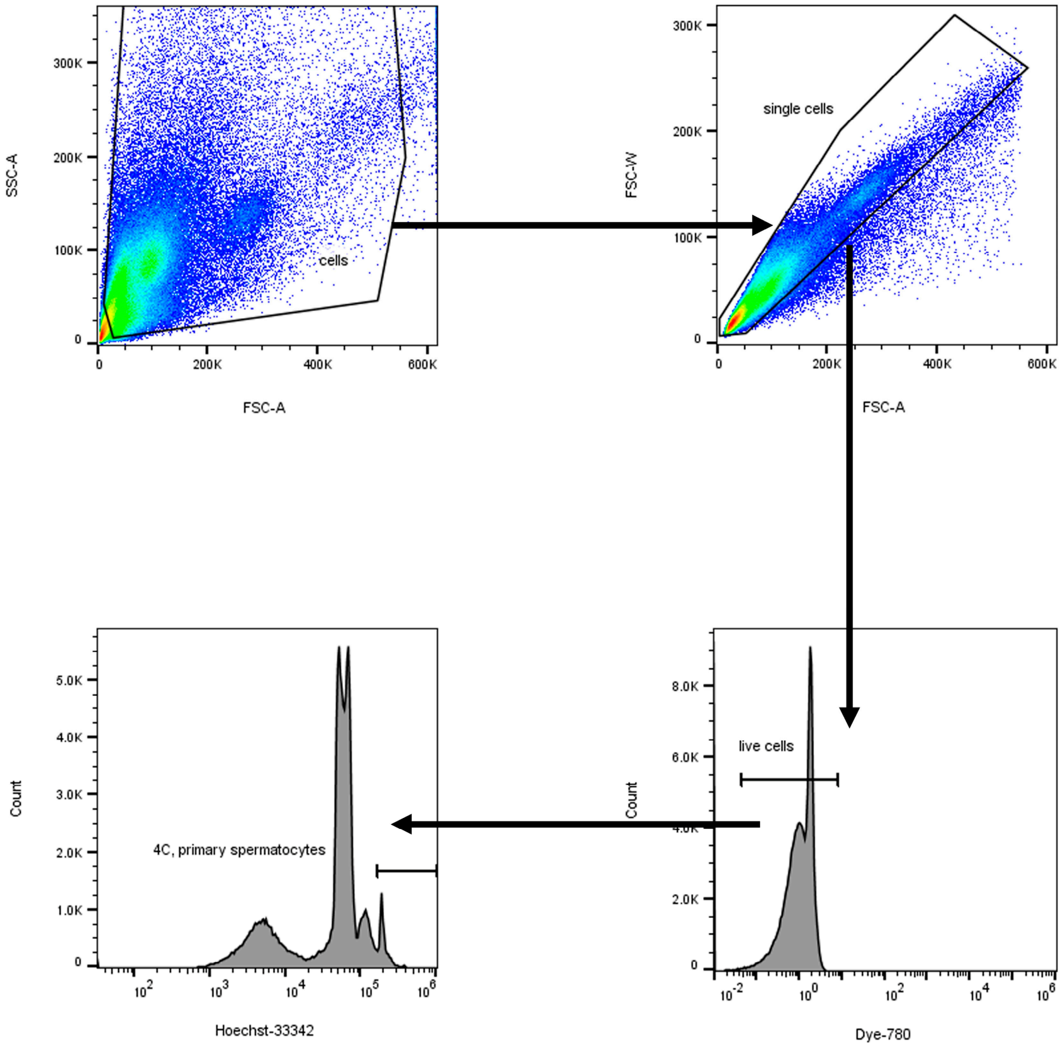

Supplementary Figure S3. Schematic diagram of the gate strategy for flow cytometric sorting of primary spermatocytes from the mouse testis. This image illustrates the gate strategy for primary spermatocytes isolation from the mouse testis.
